# Protective water curtains as wave attenuators for blast-resistant tunnels

**DOI:** 10.1038/s41598-022-24943-7

**Published:** 2022-11-28

**Authors:** Payam Keshavarz Mirza Mohammadi, Seyed Hamed Khalilpour, Hasan Parsa, Pooya Sareh

**Affiliations:** 1 Faculty of Civil, Water, and Environmental Engineering, Shahid Beheshti University, Tehran, Iran; 2 Department of Civil Engineering, Tarbiat Modares University, Tehran, Iran; 3 Department of Mechanical Engineering, Faculty of Engineering, Arak University, Arak, Iran; 4 Creative Design Engineering Lab (Cdel), Department of Mechanical and Aerospace Engineering, School of Engineering, University of Liverpool, Liverpool, L69 3GH UK

**Keywords:** Civil engineering, Mechanical engineering

## Abstract

Tunnels, as highly cost-demanding infrastructures which facilitate the transportation of people and goods, have been a target of terrorist attacks within the past few decades. The significance of the destructive impact of explosives on these structures has resulted in research on the development of blast-resistant design approaches. In this paper, water curtains are proposed as a blast-resistant system due to the established performance of water against explosives in free fields in previous studies as well as its capability to mitigate the potential incoming fire after an explosion. A parametric study was conducted for this purpose, considering the effects of curtain thickness, the distance of the curtain from the tunnel opening, and the amount of TNT charge. Accordingly, fifty-two finite element (FE) models were created in the FE package ABAQUS to investigate the performance of a water wall in a typical tunnel through the Eulerian approach to simulation. The water curtains had four different thicknesses and were located at three different distances from the reference point. TNT explosive charges were placed at the tunnel opening with four different masses. The thicker walls nearer to the tunnel opening were found to be more effective. However, the peak pressure reduction in all charges was in a desirable range of 53 to 80%. The parametric study also illustrated that the peak pressures were more sensitive to wall thickness rather than TNT charges mass and the wall distance from the explosives. We anticipate this preliminary study to be a starting point for the further development of the concept of water curtains for blast mitigation.

## Introduction

Developments in the design, construction, and utilization of underground structures due to scarcity of land areas have resulted in different applications, including various transportation and storage services. In particular, from the viewpoint of governments, there are two different objectives for investments in underground structures: (1) the transportation of goods and people, and (2) logistics for services and programs related to national defense and security. These underground structures are categorized as high-risk structures as they are generally prone to natural and man-made hazards. Many bombing attacks have occurred in recent years; examples include explosions in Oklahoma and New York City in the U.S. in 1995^1^, as well as Moscow and Saint Petersburg metro tunnel attacks in 2010 and 2017, respectively^2^. Importantly, infrastructures, and in particular high-cost tunnels, are vulnerable to such terrorist attacks. A blast is considered to be an extreme unforeseen force that can be applied to a tunnel structure, causing damage to the tunnel and its facilities, as well as the fatality of people.

Due to the importance of resilience and safety in such scenarios, researchers have conducted various analytical, numerical, and experimental studies to understand the blast behavior and develop strategies and methods to reduce the blast waves propagating in tunnels. Kim et al.^3^ proposed an empirical formulation to predict the deformation of offshore blast walls subjected to hydrocarbon explosions, where the empirical formulas were derived by a regression analysis of simulation results from the software LS-DYNA. Wahab and Mazek^4^ used sandwich panels to resist explosive events by energy dissipation through large plastic deformations. Hadianfard and Farahani^5^ investigated the cross-sectional properties of steel columns subjected to blast loadings, where a numerical nonlinear time history analysis using the software ANSYS demonstrated the significance of elastic–plastic properties of cross-sections. Through measuring vibrations in a tunnel excavation, Ozacar^6^ proposed a new method to protect shallow tunnels against blast-induced vibrations.

The concept of wall or barrier application against explosions has been a popular field of study, especially in recent years. Altunisik et al.^7^ explored the effects of concrete strength and openings in infill walls against TNT explosives. Meng et al.^8^ studied the rigid wall effect against underwater explosions by bubble pulsation monitoring. Koksal and Karaca^9^ examined reinforcement retaining walls in a blast-induced ground motion to determine the associated dynamic response.

The blast in the tunnel is a complex phenomenon due to various factors including structural discontinuities and the reflections of blast waves. While, in general, the physical experimentation of such phenomena is extremely costly, these difficulties require further in-depth investigations due to their high practical importance. To fill this knowledge gap and develop effective mitigation strategies, much numerical research has been conducted to estimate the blast loading and its effects on tunnels. For instance, Qian et al.^10^ numerically studied the performance of a utility tunnel against ground surface explosions by simulating associated damages and responses.

Protecting existing tunnels by reducing the effective blast wave strength has been another field of interest recently. A straightforward technique for enhancing the serviceability of structures is to provide a blast barrier as a wall in front of the propagated blast wave. This protective wall is placed at a distance to protect a certain space or a part of a structure from the adverse effects of an external explosion. Hence, a portion of the explosive energy is reflected, and the peak pressure is reduced. Both experimental and numerical results have illustrated that a blast wall can effectively protect structures from explosions. Rose et al.^11^ prepared a one-tenth scale blast wall and placed it at an appropriate distance from the structure. Compared with the case when the wall was absent, the recorded pressure–time results demonstrated the effectiveness of a blast wall. Bogosian and Piepenburg^12^ conducted experiments intending to investigate the effect of the mass of the concrete panel wall rather than its strength on the attenuation of the wave energy. They concluded that almost the same reduction was observed in all cases with different wall materials including soil, concrete, and water. Ngo et al.^13^ performed a parametric study on the effects of distance and wall height on peak pressure reduction. They presented a series of approximate formulas to predict the maximum reflected pressure behind the wall.

Reinforced concrete is extensively used in the construction of protective walls due to its capability in energy absorption under high pressures, making it particularly useful in many disaster mitigations scenarios such as explosions. It is widely used in many engineering projects all over the world because of its associated speed of construction, high quality, and cost-effectiveness. Hajek and Foglar^14^ investigated the peak overpressure reduction using concrete blast barriers. A computer modeling in the software LS-DYNA was calibrated based on experimental data. Besides, the effectiveness of different barrier arrangements was evaluated and analyzed comparatively. Hajek et al.^15^ focused on the resistance of plain and fiber reinforced concrete elements under blast loading using an ultra-high-speed camera. Groethe et al.^16^ examined the detonation of hydrogen gas through full-scale experiments. The blast scenarios included free field, tunnel, partial confinement, and blast wall experiments. The 4 m tall and 10 m wide RC walls at a distance of 4 m from the explosive were examined in these experiments.

In addition to rigid concrete materials, the favorable properties of non-rigid materials as blast barriers have led to the development of a new approach to blast overpressure reductions involving the attenuation of the shock wave energy through the deformation of the barrier. Aleyassin et al.^17^ studied the response of cellular materials subjected to air blasts, concluding that these systems could either enhance or attenuate the transmitted shock. Keshavarz et al.^18,19^ evaluated the performance of water and concrete walls under blast loading considering various physical and mechanical parameters. They reported the strength and the fracture energy of concrete as the most crucial factors affecting the efficiency of the walls. Moreover, they optimized flat steel explosion-proof doors by the application of inner stiffening profiles^20^. They also used barriers in different configurations and shapes^21^ and sacrificial protective walls composed of lightweight concrete blocks^22^ to attenuate blast waves in tunnels.

Last but not least, water is another material with a significant effect on blast wave mitigation. Sugiyama et al.^23^ examined the barrier material effect on blast overpressure. They examined a mixture of water, polystyrene, and spherical pentolite, with the water volume fraction as the study variable. Chen et al.^24^ proposed a water wall for blast mitigation with advantages including low cost and ease of implementation. They performed a range of small-scale experiments within an explosion chamber and carried out a simulation-based parametric study with the standoff distance and barrier dimensions as variables. They concluded that both variables are effective in the reduction of the overpressure value, with higher and nearer walls resulting in better performance. Chen et al.^25^ filled polyethylene containers with water and prepared a simple water wall. They set a detonation charge at different wall distances and a steel chest with pressure gauges on the opposite side. Then, a numerical model in the software ANSYS was developed and verified by experimental data. A parametric study with water/charge, wall height, wall distance, and water barrier thickness was performed, and the optimum values of these parameters were determined.

Whilst prior studies have mostly dealt with the application of concrete walls for protection against explosions in both open and confined spaces, the investigation of water walls has been limited to open-space explosion scenarios. In this study, a three-dimensional (3D) numerical model is established in the software ABAQUS to investigate the capability of water curtains as protective barriers in tunnel structures against TNT explosions. To this end, we have conducted a parametric study to examine the influence of different variables on the peak pressure of the ongoing blast wave to the safe surface of investigation. Furthermore, two preliminary verification studies for outdoor and indoor explosions are carried out before examining the main models, as described in the next section.

## Preliminary verification studies

Given that experiments involving explosions, specifically inside a tunnel, are extremely limited and expensive, we explored and exploited the existing literature to perform verification studies before establishing the main models for further analysis. To this end, two verification studies, namely the ‘outdoor’ explosion and the ‘indoor’ explosion, are introduced and discussed. First, the outdoor blast reported by Chen et al.^25^ is investigated and verified using their experimental and numerical results. This is the main verification study on the water simulation procedure and the corresponding responses in an explosion. Subsequently, an indoor explosion in a confined tunnel, reported by Liu et al.^26^, is simulated and verified.

### Verification of the outdoor explosion

Chen et al.^25^ used water in polyethylene containers as water barriers against blast loadings. A series of field tests were implemented besides numerical finite element simulations. The field tests demonstrated an appropriate mitigation effect of water barriers. Furthermore, the numerical results of overpressures were reported to be in good agreement with the field test results.

In this explosion scenario, the system consisted of a water wall between the TNT explosive and a wall equipped with pressure gauges to measure the overpressure reaching it. A finite element model using the CEL method was established in ANSYS-AUTODYN and verified in terms of pressure–time diagrams by the in-situ tests for the purpose of a parametric study. The TNT explosives followed the Jones-Wilkins-Lee (JWL) equation of state (EOS). The air was modeled as an ideal gas, using the gamma-law EOS, expressed by the following equation^27^:

1 $$p=\left(\gamma -1\right)\frac{\rho }{{\rho }_{0}}E$$

where $p$ is the air pressure, $\gamma$ is the heat capacity ratio, $\rho$ is the current air density, ${\rho }_{0}$ is the initial air density, and $E$ is the internal energy per unit mass. The following values were considered in this study: $\gamma =$ 1.4, ${\rho }_{0}=$ 1.225 kg/m^3^, and $E=$ 0.2068 MJ/kg. Moreover, the polyethylene container was modeled with a shock EOS as follows

2 $$p={p}_{H}+\Gamma \rho (e-{e}_{H})$$

where *p* is pressure, $\rho$ is instant density, $\Gamma$ is the Gruneisen coefficient, and ${p}_{H}$ and ${e}_{H}$ are the Hugoniot pressure and specific energy in the EOS of polyethylene calculated, respectively, from the following equations

3 $${p}_{H}=\frac{{\rho }_{0}{c}_{0}^{2}\mu (1+\mu )}{{[1-\left(s-1\right)\mu ]}^{2}}$$

4 $${e}_{H}=\frac{{p}_{H}}{2{\rho }_{0}}\left(\frac{\mu }{\mu +1}\right)$$

where ${\rho }_{0}$ and *e* are the initial density and the internal energy per unit volume of polyethylene, respectively; $\mu =\eta -1$, where $\eta$ is the ratio of density before and after disturbance; ${c}_{0}^{2}$ is the bulk sound speed; and *s* is the Hugoniot slope coefficient, which is the differential of shock velocity with respect to particle velocity. The values considered for these parameters are listed in Table 1.

**Table 1:** Material properties of polyethylene.

Property	Value
Initial density (${\rho }_{0}$)	915 kg/m^3^
Gruneisen coefficient ($\Gamma$)	1.64
Bulk sound speed (${c}_{0}$)	2460 m/s

**Figure 1:**
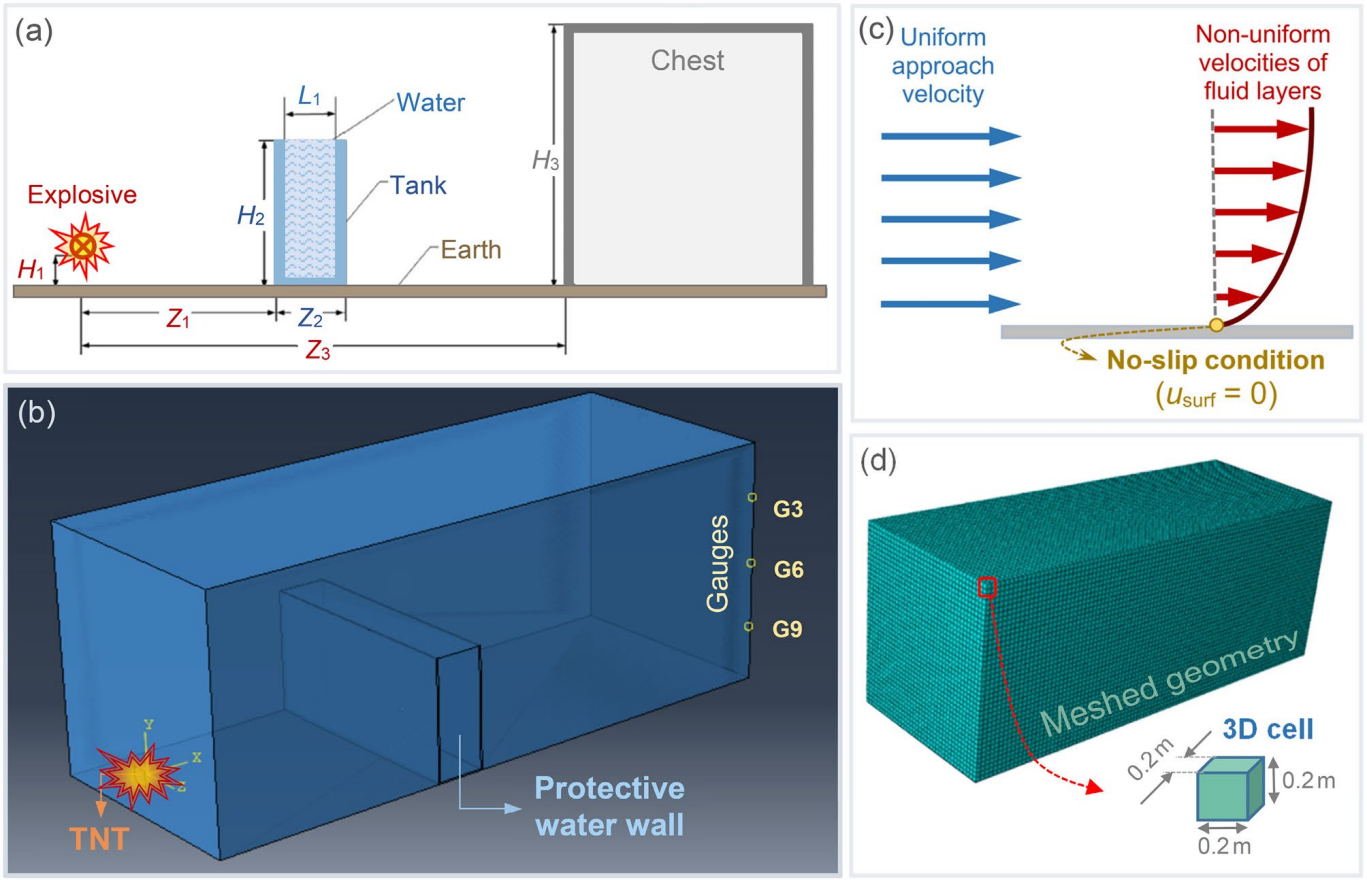
(**a**) Schematic representation of the indoor verification model (adapted from^25^). (**b**) Geometry of the verification model including the TNT charge, water wall, and pressure gauges. (**c**) No-sleep condition considered for the boundaries of the tunnel. (**d**) A mesh size of 0.2 m considered based on trial and error.

**Figure 2:**
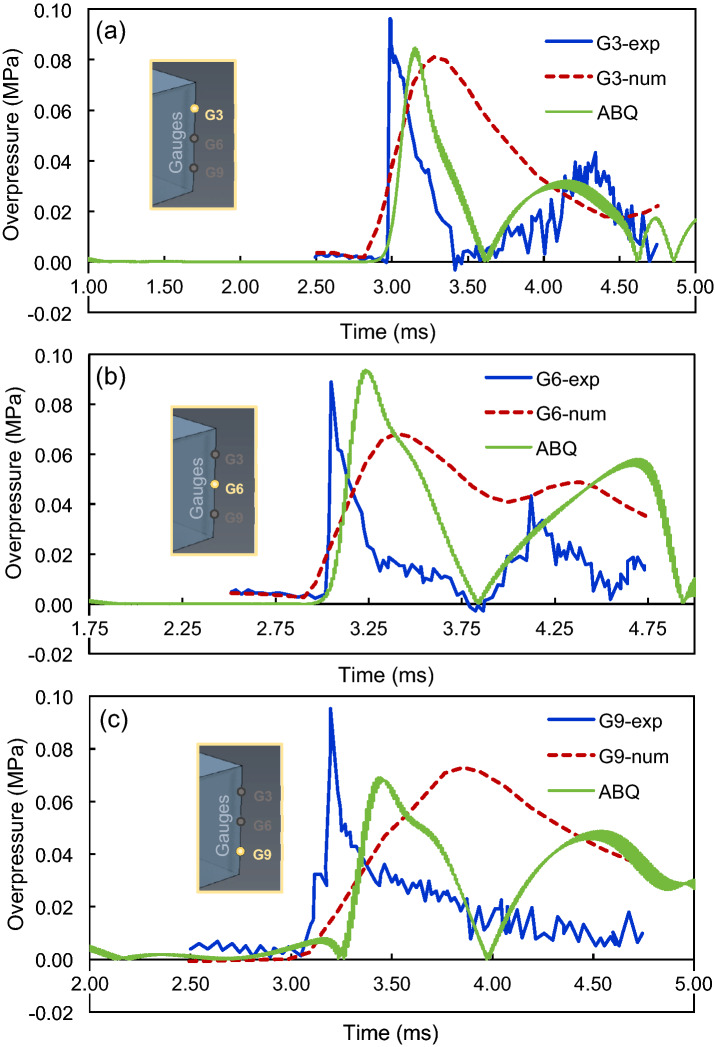
Overpressure-time diagrams of the verification model for the locations of three positioned gauges: (**a**) G3, (**b**) G6, and (**c**) G9.

As the primary material in this study, water is represented by a general polynomial form of the Mie-Gruneisen EOS in which a modification separates tension and compression^25^. Importantly, all definitions, as mentioned earlier by Chen et al.^25^, are utilized, except for the water EOS for which we used the general form of water rather than the separated form of compression and tension behavior used in the abovementioned study. The linear form of *U*_s_-*U*_p_ EOS was employed as in several other studies^28,29^. The schematic of the experiment is illustrated in Fig. 1a. The Eulerian approach to simulation, rather than the Coupled Eulerian–Lagrangian (CEL) approach, is performed in ABAQUS, with the definitions of volume fractions of TNT, water, air, and polyethylene in a void medium as shown in Fig. 1b. The TNT charge is placed at the origin of the 3D cartesian coordinate system. Three pressure gauges, namely G3, G6, and G9, are also placed at the rear end of the void. As can be seen from Fig. 1c, the boundary conditions are the same as the model in the paper. As illustrated in Fig. 1d, a mesh size of 0.2 m is considered based on trial and error, while in the model created in ANSYS by Chen et al.^25^, a smaller mesh size of 0.01 m was implemented. Since only a half of the tunnel was modeled due to its bilateral symmetry, the surface alongside the symmetrical axis is zero-velocity just in the direction perpendicular to the wave direction. With the above definitions, the overpressure-time diagrams of the model are determined from gauges G3, G6, and G9. As illustrated in Fig. 2, our numerical results from simulations in ABAQUS, labeled as ABQ, are compared with those of the experimental and numerical models of Chen et al.^25^, labeled as G-exp and G-num, respectively.

According to the diagrams, it can be seen that the peak pressure derived from ABAQUS can estimate the experimental value very closely, even better than the previously simulated model in ANSYS. The difference with the experimental results, which can be seen in both numerical models, could be due to the dynamics inherent to the analysis. The ABAQUS model predicts the pressure very closely, specifically at gauges G3 and G6 with errors of 13.2% and 6.5%, respectively; however, the G9 reading in ABAQUS showed a larger error than the numerical model of Chen et al.^25^. Furthermore, although a time delay is observed in both simulations, it is less in value and closer to the experimental data in the ABAQUS model than in the ANSYS model. Besides, a second peak pressure can be seen in the test data which is better predicted in the ABAQUS model, specifically at gauge G3.

Table 2 shows the comparison of peak times, peak pressures, and relative error values associated with the peak pressures. The results of the ABAQUS model were in good convergence with the experimental data with the relative error of peak pressures of 11.22, 5.62, and 28.57%. The ABAQUS model produced less error than the ANSYS numerical model proposed by Chen et al.^25^ at gauges G3 and G6. However, at gauge G9, both numerical models had errors of more than 20%, with a slightly larger error reported for the ABAQUS model. On the other hand, the ABAQUS model predicted the peak pressure times more accurately than the ANSYS model at all the three gauges.

**Table 2:** Numerical outputs for peak times, peak pressures, and their associated errors in the verification model obtained from different experimental and numerical studies.

Gauge	Model	Peak time (ms)	Peak pressure (MPa)	Relative error of peak pressure (%)
G3	Experimental^25^	2.98	0.098	0.00
	Numerical (ANSYS)^25^	3.38	0.080	18.37
	**Numerical (ABAQUS)**	**3.19**	**0.087**	**11.22**
G6	Experimental^25^	3.04	0.089	0.00
	Numerical (ANSYS)^25^	3.42	0.067	24.72
	**Numerical (ABAQUS)**	**3.16**	**0.094**	**5.62**
G9	Experimental^25^	3.15	0.098	0.00
	Numerical (ANSYS)^25^	3.82	0.074	24.49
	**Numerical (ABAQUS)**	**3.42**	**0.070**	**28.57**

Bold values are the results of our numerical simulations, whereas the other values are from the literature.

### Verification of the indoor explosion

**Figure 3:**
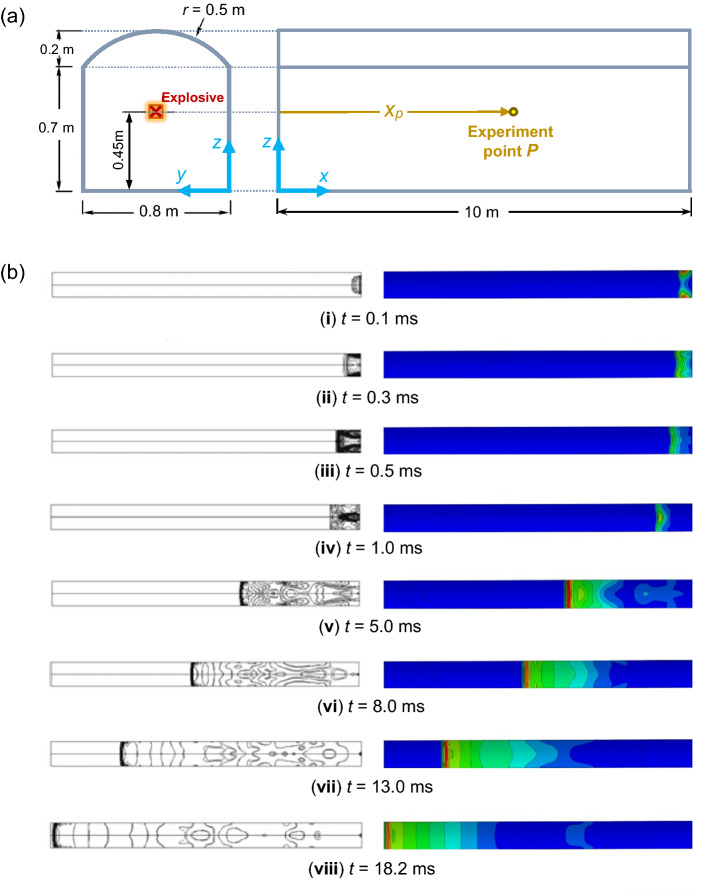
(**a**) Cross-sectional and profile views of the tunnel model (adapted from Liu et al.^26^). (**b**) Pressure contours at different time steps. Left: FEA results in LS-DYNA reported by Liu et al.^26^; right: FEA results of the ABAQUS numerical simulations of the present study.

Liu et al.^26^ investigated the blast wave propagation inside a tunnel the cross-sectional area of which is shown in Fig. 3a. The experimental results associated with the numerical simulations of their investigation were derived from a previous study by Xiao-hua and Zhang^30^. The tunnel is an underground structure with a length of 10 m. The explosive is 0.6 kg, located at a height of 0.45 m above the ground. Further details of these simulations can be found in^26^.

The simulations of TNT and air are according to the JWL EOS and ideal gas equations, respectively. The results are determined as pressure contours and pressure–time diagrams. Figure 3b illustrates the pressure contours from our FEA simulations in ABAQUS compared with those of Liu et al.^26^ simulated in LS-DYNA. According to this figure, a good conformity is observed in the blast wave location at different time steps.

**Figure 4:**
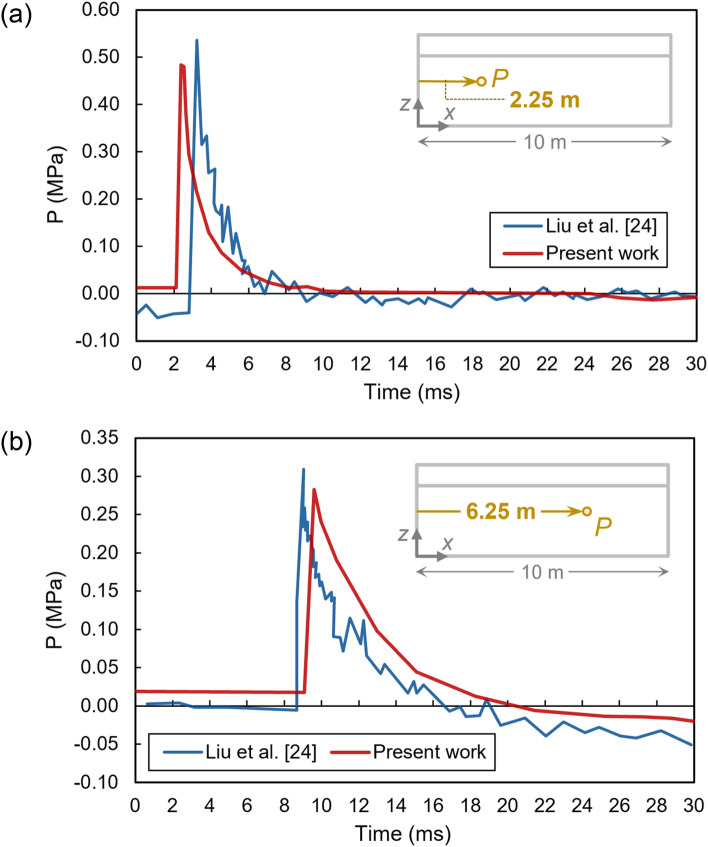
Pressure–time diagrams from our FEA simulations in comparison with those of Liu et al.^26^ at distances of (**a**) 2.25 m, and (**b**) 6.25 m, from the explosive charge.

Another comparison is with regard to time-pressure diagrams, determined at two different depths of 2.25 and 6.25 m, as depicted in Fig. 4. Similar to the previous results, a good convergence can be observed here. The peak pressures of these diagrams are listed and compared in Table 3, revealing peak pressure errors of less than 9% for both distances of 2.25 and 6.25 m. Furthermore, the experimental reaching times of the wave to the target points were 3.1 and 8.9 ms for the 2.25- and 6.25-m distances, respectively; similarly, our numerical simulation resulted in the values of 2.81 and 9.35 ms, respectively, showing errors of less than 10% for both cases.

**Table 3:** Comparison of experimental and numerical outputs for the verification model.

Distance (m)	Model	Peak pressure (MPa)	Time (ms)
2.25	Experimental	0.53	3.10
	Numerical	0.49	2.81
	Error	8.43%	9.35%
6.25	Experimental	0.31	8.90
	Numerical	0.29	9.35
	Error	8.54%	7.56%

## Numerical modeling of the main problem

A tunnel model is adopted to evaluate the performance of water curtains for the reduction of blast-induced overpressures through numerical simulations performed in ABAQUS. The details of the modeling are presented in the following subsections.

### Tunnel geometry and specifications

**Figure 5:**
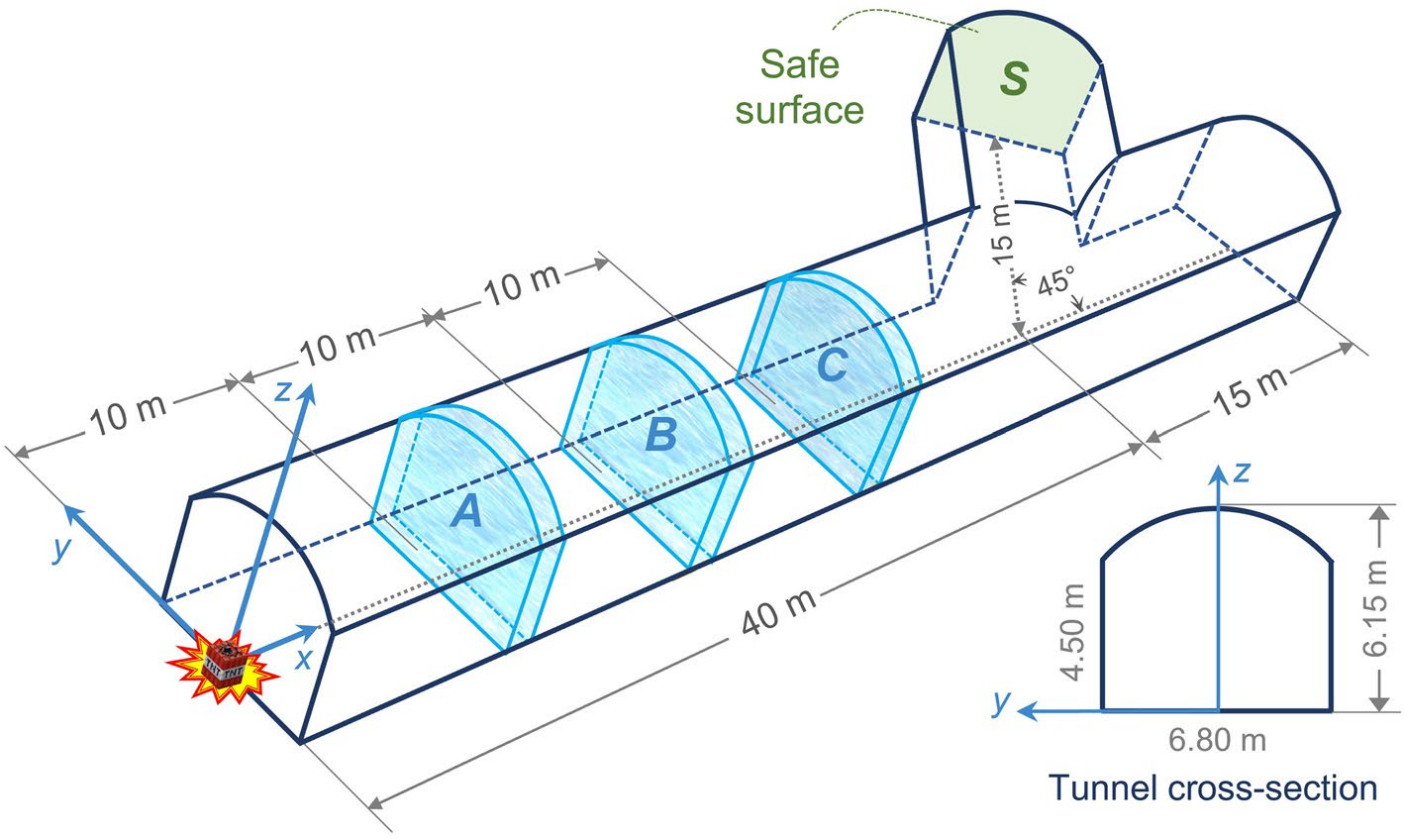
Cross-sectional and 3D views of the tunnel and the TNT charge location. Water curtains *A*, *B*, and *C* are positioned at distances of 10, 20, and 30 m from the opening, respectively.

The cross-sectional and 3D views of the tunnel are shown in Fig. 5. The total length of the tunnel is 55 m which includes a 45°-deviation of 15 m at a 40-m distance from the opening of the tunnel. The maximum height and width of the tunnel are 6.15 and 6.8 m, respectively. The surface chosen for this study is at the end of the deviation, which is called the *safe surface*, denoted by *S*. The average values of pressure at this surface are used for comparative investigations of the numerical results. The detonation charge is placed at the opening of the tunnel, as shown in Fig. 5, with four different masses of 400, 1000, 1500, and 2000 kg considered in this study. For each simulation, the volume of the TNT is calculated based on the density of the TNT explosive. As designated in Fig. 5, water curtains *A*, *B*, and *C* are placed at three different locations of 10, 20, and 30 m from the tunnel opening, respectively.

### Material properties

The defined Eulerian void consists of three materials, namely TNT, water, and air. The specifications of materials properties in ABAQUS are based on the verified data from previous studies in the literature. The air is modeled as an ideal gas as expressed by Eq. (1). In order to simulate compressible viscous and inviscid fluids, a linear *U*_s_-*U*_p_ equation of state can be defined in ABAQUS. Using the principal Hugoniot curve as a reference, the Mie Gruneisen EOS is defined. This EOS for water pressure is assumed as a function of density and internal energy per unit mass. The water is defined with the details described in “Verification of the outdoor explosion” section^25^.

Using the Jones-Wilkins-Lee (JWL) equation of state, the TNT explosive pressure can be calculated as follows^31^

5 $$p=A\left(1-\frac{\omega }{{R}_{1}\overline{\rho }}\right){e}^{-{R}_{1}\overline{\rho }}+B\left(1-\frac{\omega }{{R}_{2}\overline{\rho }}\right){e}^{-{R}_{2}\overline{\rho }}+\omega \rho {e}_{int}$$

where *A*, *B*, ${R}_{1}$, ${R}_{2}$, and $\omega$ are constants associated with the TNT explosive. Parameters *A* and *B* are related to the magnitude of pressure, $\overline{\rho }$ is the ratio of density of the explosive in the solid state to the current density, and ${e}_{int}$ is the specific internal energy at atmospheric pressure. The exponential terms in this equation demonstrate the high pressure generated during the explosion. Furthermore, the last term of this equation is a representation of the high volume of the explosion. Table 4 shows the suggested values for the parameters of Eq. (5) as given in^31^.

**Table 4:** Material properties of TNT.

Density (kg/m^3^)	Detonation wave speed (m/s)	*A* (MPa)	*B* (MPa)	$\omega$	${R}_{1}$	${R}_{2}$	Detonation energy density (kJ/kg)
1630	6930	373,800	3747	0.35	4.15	0.9	3680

In order to simulate incompressible viscous and inviscid fluids, a linear *U*_s_-*U*_p_ equation of state can be established in ABAQUS (it should be noted that if the pressure of the water affected by the shock wave is not too high, it can be considered perfectly incompressible^32^; however, because the pressure reached as high as 16 bar during the 2000 kg explosion, we also considered compressibility for accuracy and reliability purposes). Using the principal Hugoniot curve as a reference, the Mie Gruneisen EOS is defined. This EOS for water pressure is assumed to be a function of density and the internal energy per unit mass. The general form of the Mie Gruneisen equation^28^ is as follows

6 $$p-{p}_{H}=\Gamma \rho ({E}_{m}-{E}_{H})$$

where ${E}_{m}$ is the internal energy per unit mass; ${p}_{H}$ and ${E}_{H}$ are the Hugoniot pressure and specific energy, respectively; $\Gamma$ is the Gruneisen ratio calculated as $\Gamma ={\Gamma }_{0}{\rho }_{0}/\rho$, where ${\Gamma }_{0}$ is the material constant and ${\rho }_{0}$ is the reference density. The Hugoniot energy is defined using the Hugoniot pressure as follows^28^:

7 $${E}_{H}=\frac{{p}_{H}\eta }{2{\rho }_{0}}$$

where $\eta$ is the nominal compressive strain expressed as $\eta =1-{\rho }_{0}/\rho$. By substitutions and simplifications of the equations, the following relationship can be established^28^:

8 $$p={p}_{H}\left(1-\frac{{\Gamma }_{0}\eta }{2}\right)+{\Gamma }_{0}{\rho }_{0}{E}_{m}$$

By a common fit to the Hugoniot data and substituting it into Eq. (8), we obtain Eqs. (9) and (10), respectively, as follows

9 $${p}_{H}=\frac{{\rho }_{0}{c}_{0}^{2}\eta }{{(1-s\eta )}^{2}}$$

10 $$p=\frac{{\rho }_{0}{c}_{0}^{2}\eta }{{\left(1-s\eta \right)}^{2}}\left(1-\frac{{\Gamma }_{0}\eta }{2}\right)+{\Gamma }_{0}{\rho }_{0}{E}_{m}$$

where ${c}_{0}$ and *s* demonstrate the linear relationship between linear shock velocity *U*_s_ and particle velocity *U*_p_ as follows

11 $$U\mathrm{s}={c}_{0}+sU\mathrm{p}$$

where ${c}_{0}$ and *s* needs to be defined in ABAQUS, with the parameters for water defined in Table 5^28^.

**Table 5:** Material properties of water^28^.

Density (kg/m^3^)	Sound speed (m/s)	Gruneisen parameter
1000	1500	0.1

The model is an Eulerian section, in which the TNT explosive and water curtain are separated as volume fractions, and the rest of the tunnel area (void) consists of air.

### Boundary condition, mesh size, and sensitivity analysis

**Figure 6:**
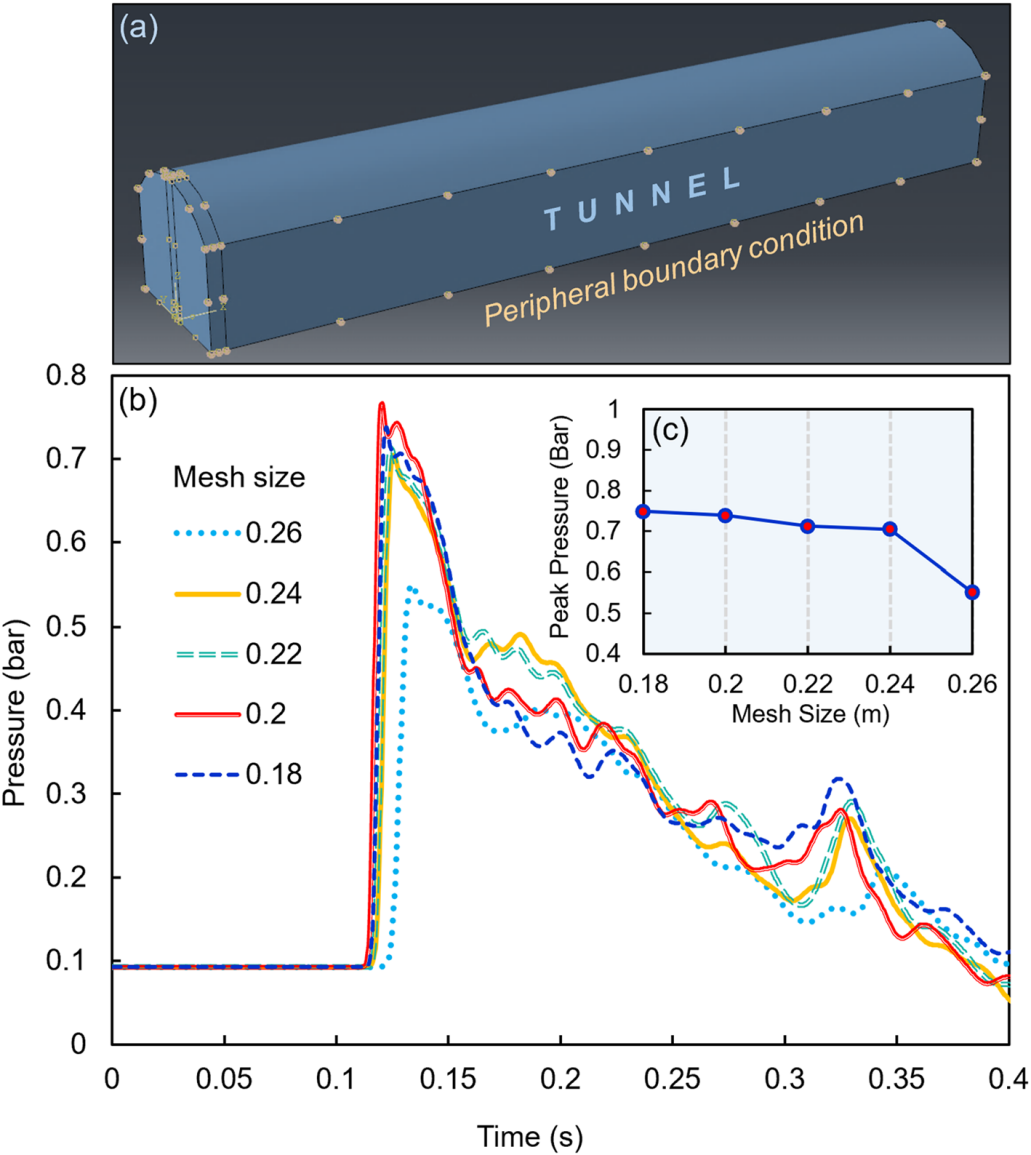
(**a**) Peripheral boundary condition of the tunnel. (**b**,**c**) Numerical results for a 400 kg TNT charge and a 0.15-m-thick water curtain located at a distance of 10 m from the tunnel opening (Part ‘**b**’ illustrates the pressure–time diagram of the sensitivity analyses for different mesh sizes, while Part ‘**c**’ shows the peak pressure plotted versus mesh size).

The periphery of the tunnel is considered to be a rock, where a boundary condition is defined rather than modeling the actual material. In other words, the secondary effect of the periphery to shake and then reflect the blast waves is not considered in the simulation to avoid the extremely high cost of calculations. Hence, as can be seen from Fig. 6a, the velocity of all surface boundaries in the three spatial directions of *x*, *y*, and *z* are fixed except for the opening surface at which the flow is free to move in or out. After examining the model and by trial and error, the mesh size of the Eulerian part is chosen to be around 0.2 m.

A sensitivity analysis is performed for a model as a case study where there are a 400 kg TNT charge and a 0.15-m-thick water curtain located at a distance of 10 m from the tunnel opening. Five different mesh sizes from 0.18 to 0.26 m are analyzed, resulting in the pressure–time diagrams illustrated in Fig. 6b. The peak pressure extracted from each analysis is presented in Fig. 6c and Table 6. The mesh size of 0.2 m is selected as the reference dimension to be compared with other mesh sizes.

**Table 6:** Results of sensitivity analyses for five different mesh sizes.

Mesh size (m)	Peak pressure (bar)	Error (%) relative to reference mesh size (0.2 m)
0.18	0.74	0.11
0.2	0.73	0
0.22	0.71	4.95
0.24	0.70	5.89
0.26	0.54	26.58

As can be seen from Fig. 6c, there is a descending pattern for the peak pressure by increasing the mesh size. This pattern has a steep slope at the beginning and the end of the diagram. In fact, the mesh size is increased until a significant drop is observed. A similar technique is used for mesh size decrease. Importantly, we can see that there is a slight difference between the results of the 0.18 m and 0.2 m mesh sizes. Hence, the 0.2 m model is selected for the present study.

### Establishment of the FEA model

This paper considers three variables: TNT charge weight, water curtain thickness, and water curtain distance from the TNT explosive. The TNT charges have four different masses of 400, 1000, 1500, and 2000 kg, while the water curtains have four different thicknesses of 0.1, 0.15, 0.2, and 0.25 m. Three different distances of 10, 20, and 30 m from the tunnel opening (or the TNT location) are considered for the water curtain. For each TNT charge, a model with no water curtain is also established for comparison purposes, resulting in a total of 52 models with labels given in Table 7. The two prefixes NW or W refer to ‘no water curtain’ and ‘water curtain’, respectively. The forthcoming numbers are the TNT charge weight in kg, the mesh size in centimeters, the curtain thickness in meters, and the distance of the water curtain from the TNT charge in meters, respectively.

**Table 7:** Labels of the FEA models and their corresponding specifications.

TNT charge (kg)	Water curtain	Curtain thickness (m)	Curtain distance (m)	Model label
400	No	–	–	NW-400-20
	Yes	0.10	30	W-400-20-0.1@30
		0.10	20	W-400-20-0.1@20
		0.10	10	W-400-20-0.1@10
		0.15	30	W-400-20-0.15@30
		0.15	20	W-400-20-0.15@20
		0.15	10	W-400-20-0.15@10
		0.20	30	W-400-20-0.2@30
		0.20	20	W-400-20-0.2@20
		0.20	10	W-400-20-0.2@10
		0.25	30	W-400-20-0.25@30
		0.25	20	W-400-20-0.25@20
		0.25	10	W-400-20-0.25@10
1000	No	–	–	NW-1000-20
	Yes	0.10	30	W-1000-20-0.1@30
		0.10	20	W-1000-20-0.1@20
		0.10	10	W-1000-20-0.1@10
		0.15	30	W-1000-20-0.15@30
		0.15	20	W-1000-20-0.15@20
		0.15	10	W-1000-20-0.15@10
		0.20	30	W-1000-20-0.2@30
		0.20	20	W-1000-20-0.2@20
		0.20	10	W-1000-20-0.2@10
		0.25	30	W-1000-20-0.25@30
		0.25	20	W-1000-20-0.25@20
		0.25	10	W-1000-20-0.25@10
1500	No	–	–	NW-1500-20
	Yes	0.10	30	W-1500-20-0.1@30
		0.10	20	W-1500-20-0.1@20
		0.10	10	W-1500-20-0.1@10
		0.15	30	W-1500-20-0.15@30
		0.15	20	W-1500-20-0.15@20
		0.15	10	W-1500-20-0.15@10
		0.20	30	W-1500-20-0.2@30
		0.20	20	W-1500-20-0.2@20
		0.20	10	W-1500-20-0.2@10
		0.25	30	W-1500-20-0.25@30
		0.25	20	W-1500-20-0.25@20
		0.25	10	W-1500-20-0.25@10
2000	No	–	–	NW-2000-20
	Yes	0.10	30	W-2000-20-0.1@30
		0.10	20	W-2000-20-0.1@20
		0.10	10	W-2000-20-0.1@10
		0.15	30	W-2000-20-0.15@30
		0.15	20	W-2000-20-0.15@20
		0.15	10	W-2000-20-0.15@10
		0.20	30	W-2000-20-0.2@30
		0.20	20	W-2000-20-0.2@20
		0.20	10	W-2000-20-0.2@10
		0.25	30	W-2000-20-0.25@30
		0.25	20	W-2000-20-0.25@20
		0.25	10	W-2000-20-0.25@10

Each analysis was performed using a four-core processor of Intel Core i9-9900K CPU @ 3.6 GHz with 64 GB RAM. The approximate computational time for the complete analysis of each model was 34 h, leading to a total computational time of 1768 h.

## Results and discussion

**Figure 7:**
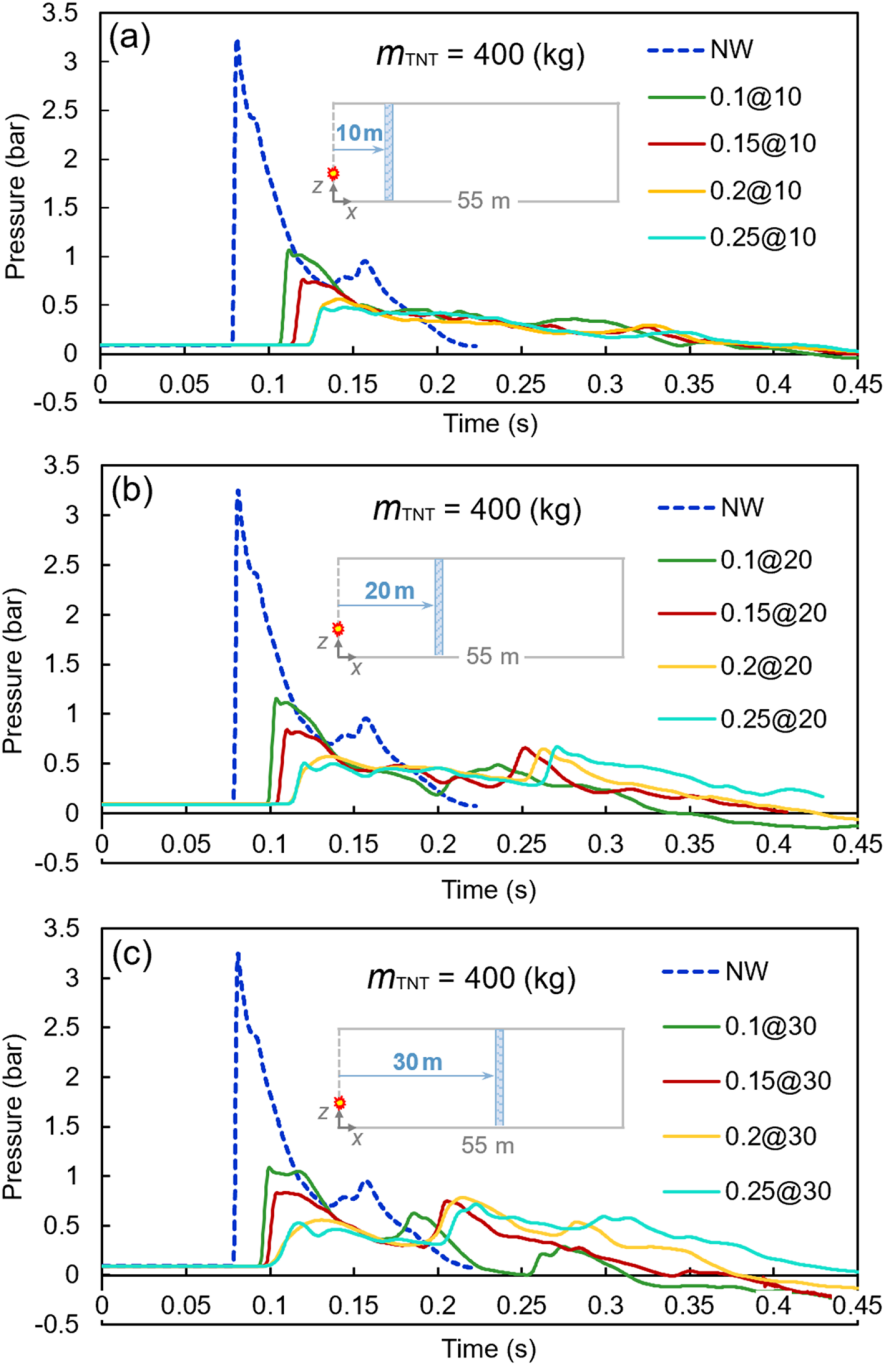
Pressure–time diagrams of the 400-kg TNT charge with different water curtains at various distances: (**a**) 10 m, (**b**) 20 m, and (**c**) 30 m.

Pressure–time diagrams were derived for each model. Figure 7 shows the pressure fluctuations of the 400 kg TNT charge with water curtains of 0.1, 0.15, 0.2, and 0.25 m thicknesses at different distances. In each Figure, the results at the same distance from the TNT charge are compared with the case in which the barrier is removed. The peak pressure of 3.24 bar in the ‘no water curtain’ case is reduced to the range of 0.48 to 1.15 bar by applying water curtain barriers.

**Figure 8:**
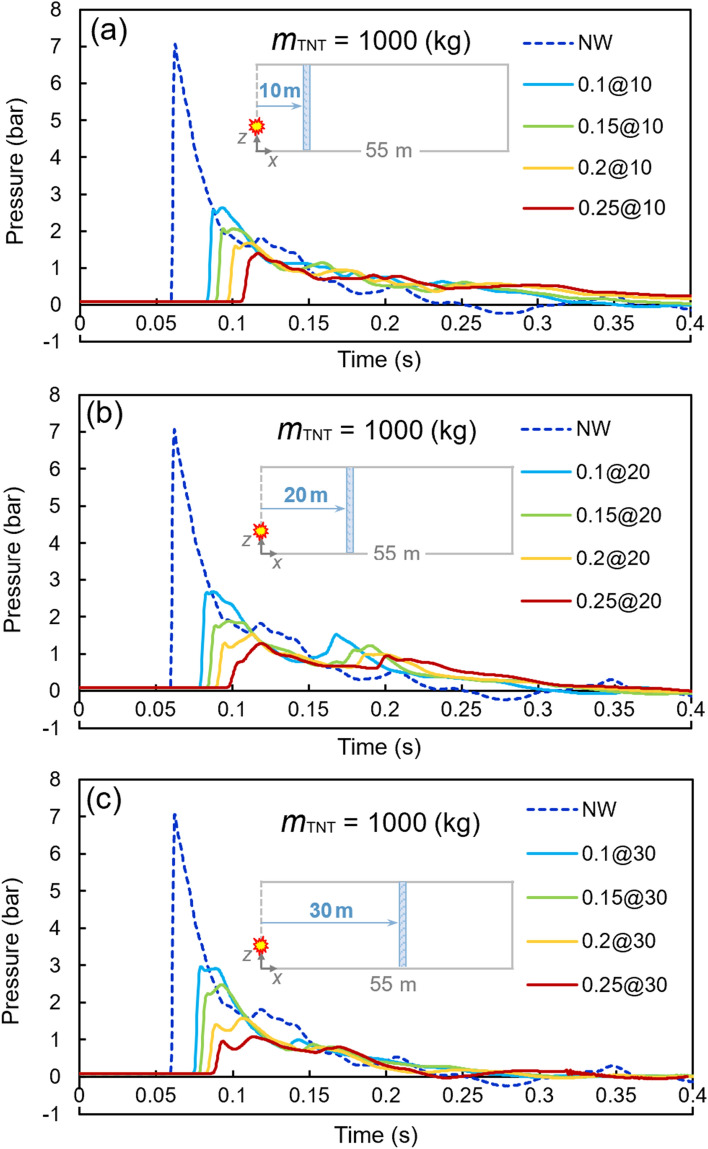
Pressure–time diagrams of the 1000-kg TNT charge with different water curtains at various distances: (**a**) 10 m, (**b**) 20 m, and (**c**) 30 m.

**Figure 9:**
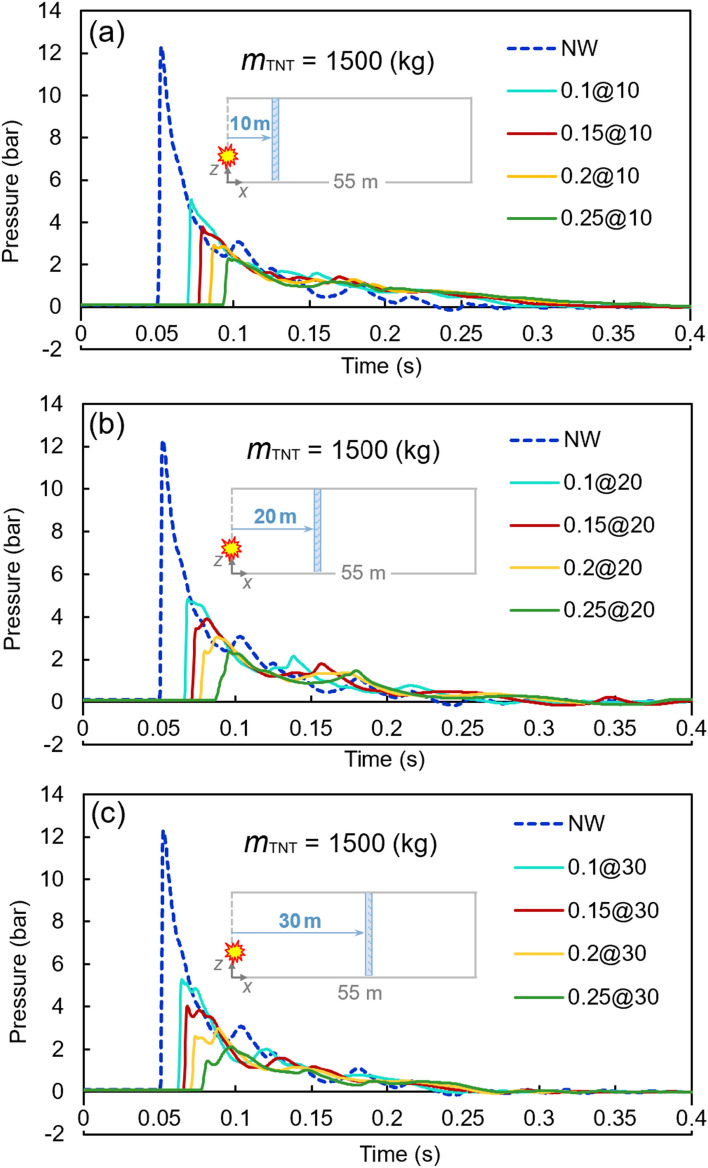
Pressure–time diagrams of the 1500-kg TNT charge with different water curtains at various distances: (**a**) 10 m, (**b**) 20 m, and (**c**) 30 m.

**Figure 10:**
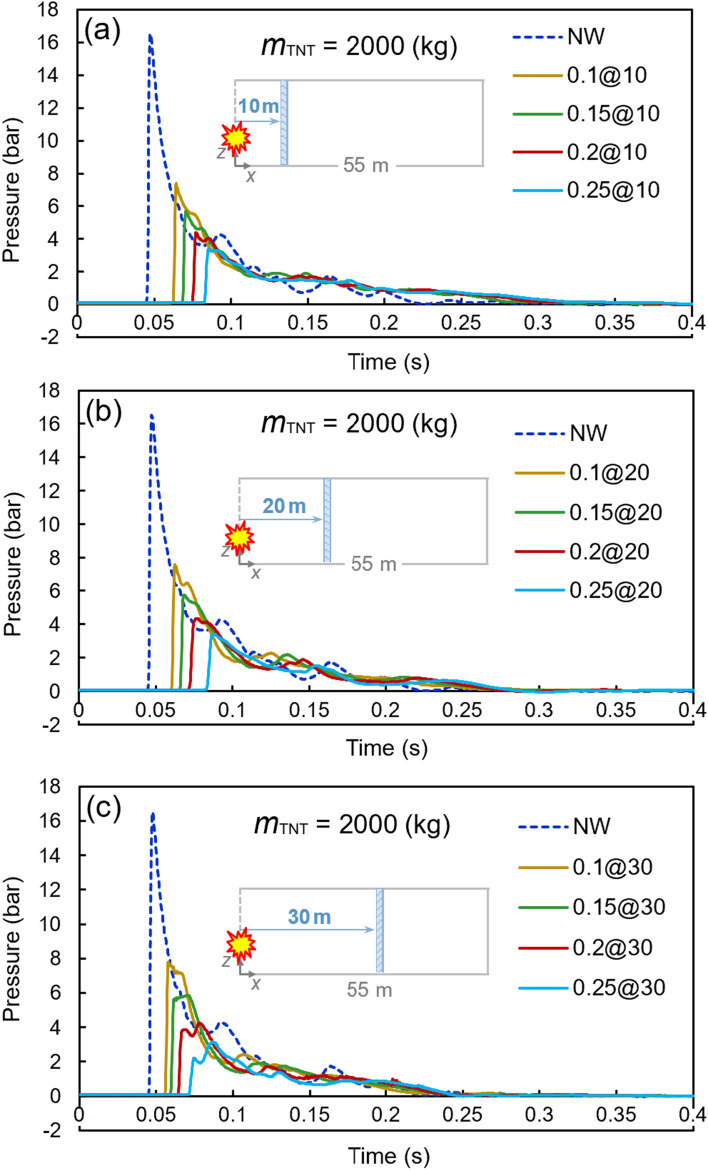
Pressure–time diagrams of the 2000-kg TNT charge with different water curtains at various distances: (**a**) 10 m, (**b**) 20 m, and (**c**) 30 m.

Figures 8, 9, and 10 present similar diagrams for the 1000, 1500, and 2000 kg TNT charges, respectively. When the 1000 kg TNT charge is considered, the peak pressure without any barrier is 7.06 bar, whilst a 0.25-m water curtain at a 30-m distance from the tunnel opening reduces the peak pressure to 1.07 bar, which is a significant decrease of around 82%. By increasing the TNT charge mass to 1500 kg, the peak pressure reaches the value of 12.29 bar. The curtain with the least thickness of 0.1 m at the distance of 20 m from the TNT charge results in a peak pressure of 4.84 bar, which is an approximately 65% reduction. The largest TNT mass used in these analyses is 2000 kg, for which the peak pressure without any barrier is 16.53 bar, while this value decreases to the range of 3.15–7.81 bar with the proposed water curtains. The details of the results associated with these diagrams are shown in Table 8.

**Table 8:** Details of peak pressures for different models.

Explosive charge	Model label	Peak pressure (bar)	Peak pressure reduction (%)
*m*_TNT_ = 400 kg	NW-400-20	3.24	0.00
	W-400-20-0.1@30	1.09	66.53
	W-400-20-0.1@20	1.15	64.43
	W-400-20-0.1@10	1.06	67.17
	W-400-20-0.15@30	0.83	74.34
	W-400-20-0.15@20	0.84	74.09
	W-400-20-0.15@10	0.76	76.40
	W-400-20-0.2@30	0.78	75.82
	W-400-20-0.2@20	0.65	79.96
	W-400-20-0.2@10	0.56	82.64
	W-400-20-0.25@30	0.71	77.97
	W-400-20-0.25@20	0.66	79.39
	W-400-20-0.25@10	0.48	85.28
*m*_TNT_ = 1000 kg	NW-1000-20	7.06	0.00
	W-1000-20-0.1@30	2.96	58.04
	W-1000-20-0.1@20	2.66	62.23
	W-1000-20-0.1@10	2.63	62.70
	W-1000-20-0.15@30	2.48	64.84
	W-1000-20-0.15@20	1.88	73.31
	W-1000-20-0.15@10	2.06	70.78
	W-1000-20-0.2@30	1.57	77.71
	W-1000-20-0.2@20	1.52	78.46
	W-1000-20-0.2@10	1.67	76.27
	W-1000-20-0.25@30	1.07	84.72
	W-1000-20-0.25@20	1.27	81.93
	W-1000-20-0.25@10	1.39	80.28
*m*_TNT_ = 1500 kg	NW-1500-20	12.29	0.00
	W-1500-20-0.1@30	5.29	56.98
	W-1500-20-0.1@20	4.84	60.64
	W-1500-20-0.1@10	5.07	58.70
	W-1500-20-0.15@30	4.03	67.18
	W-1500-20-0.15@20	3.89	68.29
	W-1500-20-0.15@10	3.79	69.12
	W-1500-20-0.2@30	2.97	75.82
	W-1500-20-0.2@20	3.05	75.14
	W-1500-20-0.2@10	2.93	76.15
	W-1500-20-0.25@30	2.12	82.72
	W-1500-20-0.25@20	2.37	80.72
	W-1500-20-0.25@10	2.29	81.38
*m*_TNT_ = 2000 kg	NW-2000-20	16.53	0.00
	W-2000-20-0.1@30	7.81	52.74
	W-2000-20-0.1@20	7.61	53.96
	W-2000-20-0.1@10	7.39	55.25
	W-2000-20-0.15@30	5.87	64.46
	W-2000-20-0.15@20	5.74	65.22
	W-2000-20-0.15@10	5.69	65.54
	W-2000-20-0.2@30	4.24	74.30
	W-2000-20-0.2@20	4.35	73.66
	W-2000-20-0.2@10	4.39	73.42
	W-2000-20-0.25@30	3.15	80.95
	W-2000-20-0.25@20	3.41	79.33
	W-2000-20-0.25@10	3.40	79.42

Using a water curtain as a barrier in front of the blast waves reduced the peak pressure significantly. A 400 kg TNT produced a peak pressure of 3.24 bar. A curtain with a thickness of 0.25 m at a 10 m distance from the explosive reduced the peak pressure to 0.48 bar, i.e. an 85% reduction approximately. This was the maximum reduction among all different cases. With the TNT charge of 2000 kg and a 0.1-m barrier at a 30 m distance from the charge, the peak pressure decreased from 16.53 (no water barrier) to 7.81 bar, which is the minimum reduction among all models with a value of around 53%. It should also be noted that the reduction ranges are 64–85, 58–84, 57–83, and 53–81% for the TNT charges of 400, 1000, 1500, and 2000 kg, respectively. The lower and upper reduction limits decreased slightly by increasing the TNT charge, which shows a lower level of dependence of that reduction rate on the TNT charge.

Increasing the curtain thickness, as expected, reduced the peak pressure effectively. For instance, using a TNT charge of 2000 kg and a curtain barrier at 30 m from the charge resulted in peak pressures of 7.81, 5.87, 4.24, and 3.15 bar for the curtain thicknesses of 0.1, 0.15, 0.2, and 0.25, respectively. This trend is also seen in other cases, revealing the direct dependence of the curtain thickness on peak pressure reduction.

The distance of the curtain from the TNT charge also resulted in changes in the peak pressure. Almost for all the models, the curtain at a distance of 10 m demonstrated better performance, while the distances of 20 and 30 m came next, respectively. For example, using a 1500 kg TNT charge and the curtain of 0.15 m thickness produced peak pressures of 4.03, 3.89, and 3.79 bar for the distances of 30, 20, and 10 m of barriers, respectively. However, the variations are different for other models. One can conclude that the distance of the water curtain plays a significant role in the value of the peak pressure.

**Figure 11:**
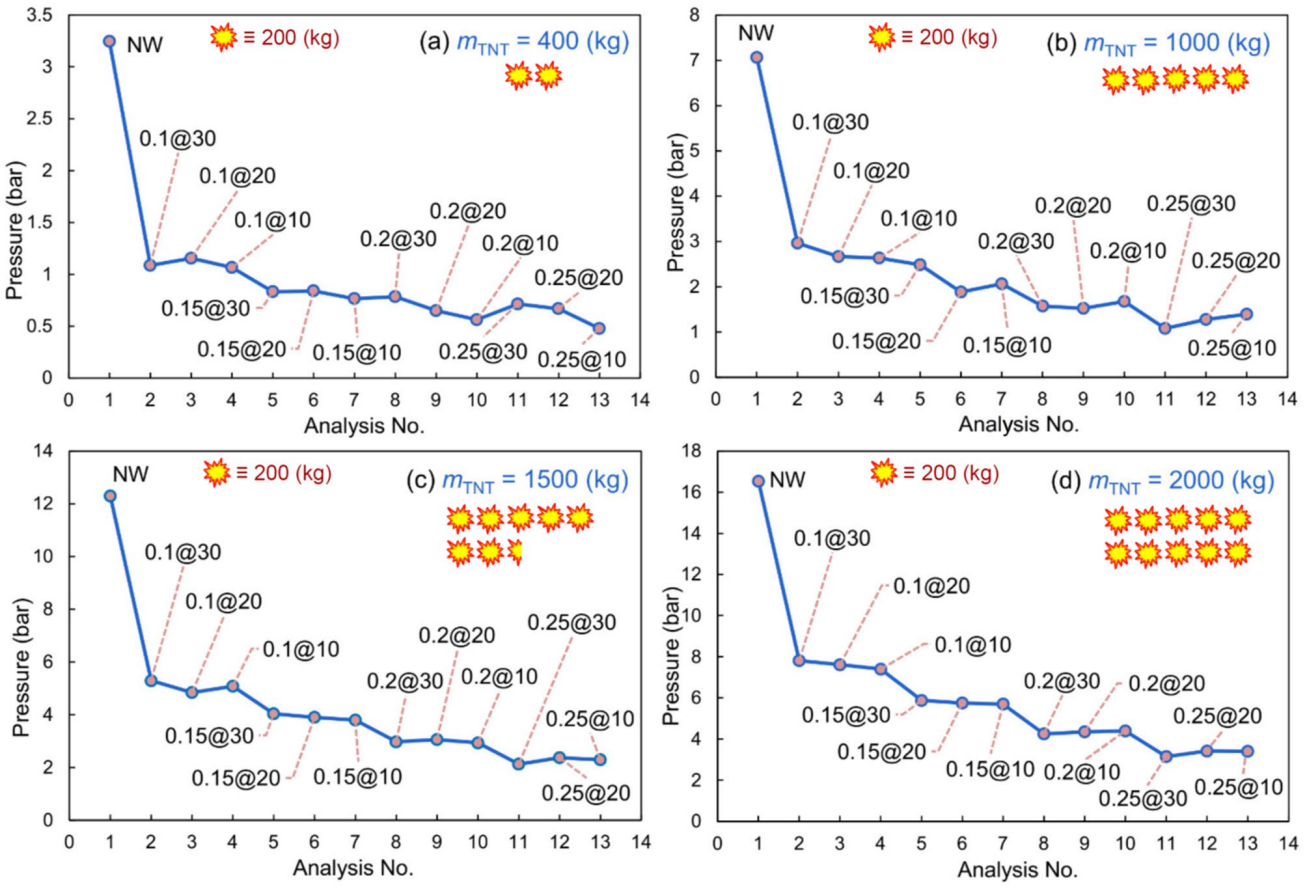
Peak pressures of different models for various TNT charges: (**a**) 400 kg, (**b**) 1000 kg, (**c**) 1500 kg, (**d**) and 2000 kg.

According to the above results, the followings are noted:

I. The significant performance of water curtains was observed. More specifically, in all analyzes for different TNT charges, the peak pressure decreased with the increase of curtain thickness.
II. In the 400 kg TNT charge models, a secondary peak of pressure was observed after the first (and major) peak. For this TNT charge, a part of ongoing waves was blocked by the curtain and reduced in speed dramatically, while the remaining waves propagated and turned back after being blocked by the end surfaces of the tunnel. These reverted waves collided with the moving-forward low-speed waves and reflected again to the end surfaces, leading to the secondary peak of the diagrams. This secondary peak was lower when the curtain was closer to the tunnel opening because the waves could flow back into the air.
III. When the TNT charge increased from 400 kg to the maximum of 2000 kg, the process of secondary peak generation was barely observed. This might be related to the higher speed of the ongoing wave due to the intense detonation of the charges. When the waves collided with the curtain, they could easily break the curtain and go through the end surfaces at a relatively high speed without any confinement of the propagated waves.
IV. For models with 400, 1000, 1500, and 2000 kg TNT, the peak pressures were reduced by 64–85%, 58–84%, 57–82%, and 63–80%, respectively. This illustrates the favorable performance of water barriers against blast waves.
V. The curtain at the 10-m distance from the tunnel opening had the best performance compared to those at the 20- and 30-m distances. However, in a few cases, the model with a 20 m distance from the opening was superior; this reveals that the nearer the curtain to the opening, the more reduction in peak pressure, which is related to the vicinity to the opening and the conduction of waves to the outside environment.
VI. Although better performance in overpressure reduction was observed for the curtains with higher thicknesses, it might not be an optimal solution operationally to use such high volumes of water.
VII. In Fig. 11, the abovementioned comparisons can be further noticed graphically. Approximately uniform variations in the performance of models, as well as superior performance of nearer and thicker water curtains, can be observed. Almost a similar pattern is observed in all the figures, demonstrating the better performance of the water curtains closer to the explosives. Besides, a slight decrease can be seen in all diagrams by increasing the curtain thickness. However, in comparison with the case that is bereft of water curtains, increasing the thickness might not be viable for operational works, because the implementation of the curtains with heavier weights would impose additional complexities.

**Figure 12:**
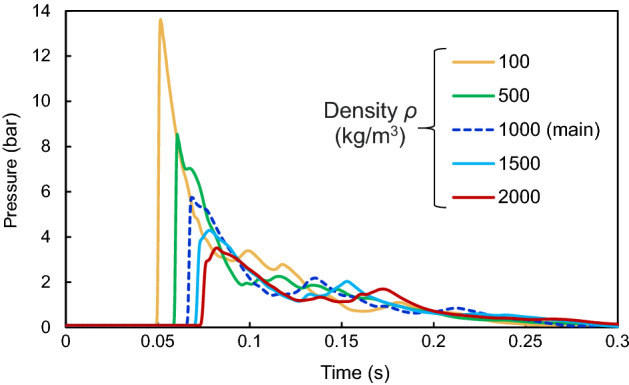
Pressure–time diagrams of model W-2000-20-0.15@20 as a function of the density *ρ*.

**Figure 13:**
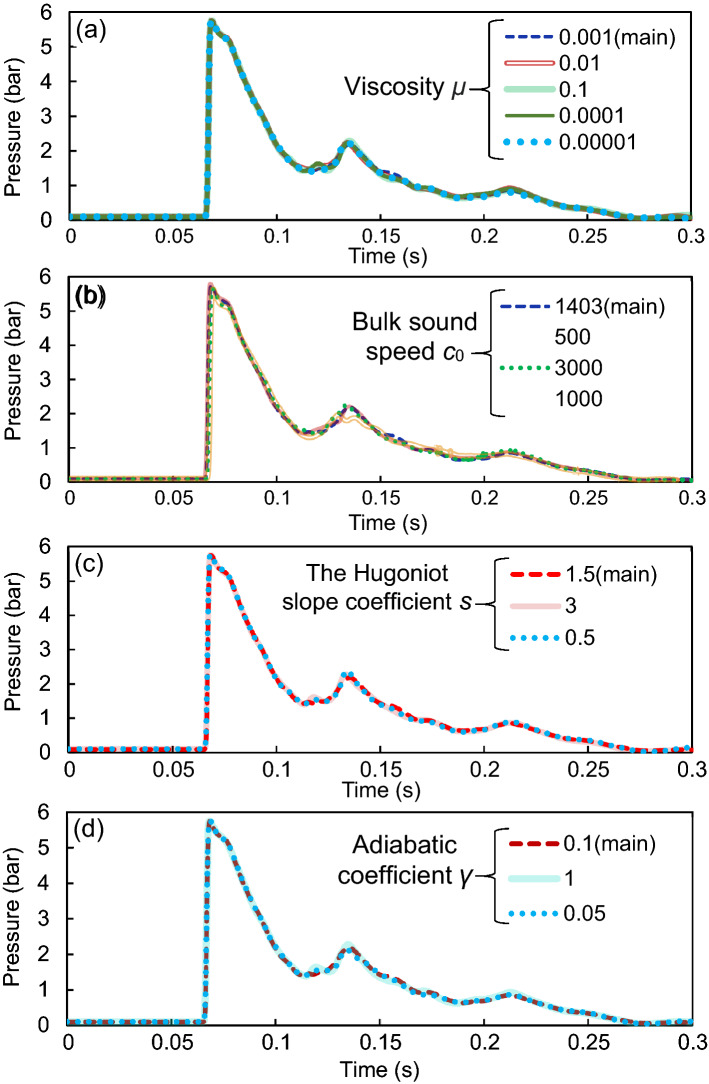
Pressure–time diagrams of model W-2000-20-0.15@20 as a function of various properties of water: (**a**) viscosity *μ*, (**b**) bulk sound speed *c*_0_, (**c**) the Hugoniot slope coefficient *s*, and (**d**) adiabatic coefficient *γ*.

The final investigation of this paper is to examine the effect of defined parameters of water in ABAQUS for simulations at the current blast loading. In this regard, five parameters of water including density *ρ*, viscosity *μ*, bulk sound speed *c*_0_, the Hugoniot slope coefficient *s*, and the adiabatic coefficient *γ* are changed individually in a randomly selected model W-2000–20-0.15@20. This model contains a water curtain with a thickness of 0.15 m located at a 20-m distance from the 2000 kg TNT charge. Figures 12 and 13 present the pressure–time diagrams in terms of these five parameters of water.

As can be observed from these figures, density is the key property of water in this specific problem of reducing blast wave energy. The more the density of the water, the more reduction in the peak pressure of the blast wave. When the density is increased from 1000 to 2000 kg/m^3^, the peak pressure is decreased from 5.7 to 3.7 bar which is a significant reduction of about 57%. Decreasing the density to 100 kg/m^3^ results in a peak pressure of 13.9 bar. However, it should be noted that other parameters that produced no effect on peak pressure reduction might be effective in different problems.

**Figure 14:**
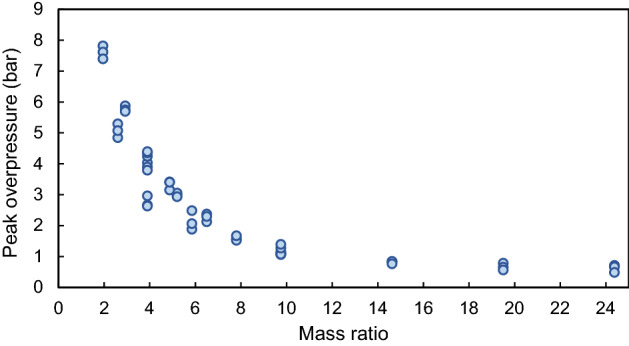
Effect of mass ratio on peak overpressure reduction.

Finally, an investigation is performed on the mass ratio of water to explosive to examine whether there is a relationship between the mass ratio and the peak overpressure reduction. In this regard, the diagram of peak overpressure versus mass ratio is depicted in Fig. 14. According to this figure, a reduction trend can be observed by increasing the mass ratio. More specifically, the peak overpressure reduction rate is relatively high for mass ratios between 1 and 8, while it decreases considerably after the mass ratio of around 8. As a result, this parameter can also be considered an effective parameter in peak pressure reduction.

## Conclusions

In this paper, we examined the performance of water curtains against blast loading in tunnels. A numerical finite element model was created for this purpose, where a parametric study of the performance of water curtains was conducted. The variables studied in this study were the TNT charge weight, the thickness of the curtain, and the distance of the curtain from the detonation charge. The results demonstrated the reliable performance of water as a curtain to resist TNT blast loads. The parametric study also revealed the superior performance of thicker curtains, as well as the curtains closer to the opening of the tunnel. In the best case of the worst scenario, i.e. the 2000 kg TNT charge, the peak pressure of the reference surface reached from 16.53 to 3.15 bar, which is equivalent to around 81% reduction of pressure waves. This was related to the 0.25-m thick curtain at the 30-m distance from the explosives. However, in other scenarios, the nearer curtains had a better performance, specifically those located at 10 m from the charge. This study demonstrated that the density of water plays an essential role to achieve the goal of this study. Also, due to the dangers of fires after explosions, and on the other hand, the presence of manpower in control rooms and construction spaces, using this mechanism could reduce damage to people, facilities, and structures. Hence, engineers in practical and operational works can establish a system for creating water curtains to protect confined structures against terrorist attacks and alleviate the fatality and high damage cost of special structures or infrastructures.

## Data Availability

The datasets used and/or analysed during the current study are available from the corresponding author on reasonable request.

## References

[1] Mosalam KM, Mosallam AS (2001). Nonlinear transient analysis of reinforced concrete slabs subjected to blast loading and retrofitted with CFRP composites. Compos. Part B Eng..

[2] Chaudhary RK, Mishra S, Chakraborty T, Matsagar V (2019). Vulnerability analysis of tunnel linings under blast loading. Int. J. Prot. Struct..

[3] Kim DK, Ng WCK, Hwang O (2018). An empirical formulation to predict maximum deformation of blast wall under explosion. Struct. Eng. Mech..

[4] Wahab MM, Mazek SA (2016). Performance of double reinforced concrete panel against blast hazard. Comput. Concr..

[5] Hadianfard MA, Farahani A, B-Jahromi A (2012). On the effect of steel columns cross sectional properties on the behaviours when subjected to blast loading. Struct. Eng. Mech..

[6] Ozacar V (2018). New methodology to prevent blasting damages for shallow tunnel. Geomech. Eng..

[7] Altunışık AC, Önalan F, Sunca F (2021). Effects of concrete strength and openings in infill walls on blasting responses of RC buildings subjected to TNT explosive. Iran. J. Sci. Technol. Trans. Civ. Eng..

[8] Meng L, Huang RY, Qin J, Wang JX, Liu LT (2020). Study on the influence of rigid wall surface on the bubble characteristics of underwater explosion. J. Phys. Conf. Ser..

[9] Köksal O, Zeki K (2020). Influence of blast-induced ground motion on dynamic response of reinforcement retaining walls. J. Struct. Eng..

[10] Qian H, Zong Z, Wu C, Li J, Gan L (2021). Numerical study on the behavior of utility tunnel subjected to ground surface explosion. Thin-Walled Struct..

[11] Rose TA, Smith PD, Mays GC (1995). The effectiveness of walls designed for the protection of structures against airblast from high explosives. Proc. Inst. Civ. Eng. Struct. Build..

[12] Bogosian, D. & Piepenburg, D. Effectiveness of frangible barriers for blast shielding. In *17th Int. Symp. Mil. Asp. Blast Shock* 78–85 (2002).

[13] Ngo, T., Nguyen, N. & Mendis, P. An Investigation on the effectiveness of blast wall and blast-structure interaction. In *51th Conf. Dev. Mech. Struct. Mater.* 957–961 (2004).

[14] Hájek R, Foglar M (2014). The reduction of peak overpressure using concrete blast barriers. WIT Trans. Built. Environ..

[15] Hajek R, Kovar M, Foglar M, Pachman J, Štoller J (2015). Field testing of concrete members subjected to contact and adjacent blast. Adv. Mater. Res..

[16] Groethe M, Merilo E, Colton J, Chiba S, Sato Y, Iwabuchi H (2007). Large-scale hydrogen deflagrations and detonations. Int. J. Hydrogen Energy.

[17] Aleyaasin M, Harrigan JJ, Reid SR (2015). Air-blast response of cellular material with a face plate: An analytical-numerical approach. Int. J. Mech. Sci..

[18] Keshavarz, M. M. P., Khalilpur, H. & Parsa, H. Performance evaluation of water walls under specific loading in closed mineral spaces. In *6th Int. Conf. Res. Sci. Eng., *vol. 6 (Kasem Bundit University, 2021).

[19] Keshavarz, M. M. P., Khalilpur, H. & Parsa, H. Performance evaluation of concrete walls under specific loading, considering physical and mechanical parameters. In *9th Int. Conf. Innov. Res. Eng. Sci.* (Georgian International Academy of Sciences and Studies, 2021).

[20] Khalilpur, H., Keshavarz, M. M. P. & Parsa, H. Flat steel resistant doors’ optimization by employing inner stiffening profiles in industrial spaces. In *6th Int. Conf. Res. Sci. Eng.*, vol. 6 (Kasem Bundit University, 2021).

[21] Eslami M, Keshavarz MMP, Khalilpour H, Parsa H, Kodur V (2022). Experimental and numerical investigation of blast wave attenuation by using barriers in different configurations and shapes. J. Struct. Eng..

[22] Keshavarz Mirza Mohammadi P, Khalilpour SH, Parsa H, Sareh P (2022). Computational performance evaluation of sacrificial protective walls composed of lightweight concrete blocks: A parametric study of blast loads in a tunnel. Mech. Adv. Mater. Struct..

[23] Sugiyama Y, Homae T, Wakabayashi K, Matsumura T, Nakayama Y (2014). Numerical simulations on the attenuation effect of a barrier material on a blast wave. J. Loss Prev. Process. Ind..

[24] Chen L, Zhang L, Fang Q, Mao YM (2015). Performance based investigation on the construction of anti-blast water wall. Int. J. Impact Eng..

[25] Chen L, Fang Q, Zhang L, Zhang Y, Chen W (2016). Numerical investigation of a water barrier against blast loadings. Eng. Struct..

[26] Liu J, Qiushi Y, Jun W (2009). Analysis of blast wave propagation inside tunnel. Trans. Tianjin Univ..

[27] Yang G, Wang G, Lu W, Yan P, Chen M (2019). Damage assessment and mitigation measures of underwater tunnel subjected to blast loads. Tunn. Undergr. Space Technol..

[28] Avachat S, Zhou M (2017). Novel experimental and 3D multiphysics computational framework for analyzing deformation and failure of composite laminates subjected to water blasts. Int. J. Impact Eng..

[29] Ahmadzadeh M, Saranjam B, Hoseini Fard A, Binesh AR (2014). Numerical simulation of sphere water entry problem using Eulerian–Lagrangian method. Appl. Math. Model..

[30] Li J, Bian X, Zhang L (2006). Numerical simulation of blast wave propagation in tunnel compared with experiment data. Shanxi Archit..

[31] Larchera, M. & Casadeia, F. *Explosions in Complex Geometries—A Comparison of Several Approaches*, vol. 1. 10.1260/2041-4196.1.2.169 (2010).

[32] Barus, C. The compressibility of liquids (No. 92) (US Government Printing Office, 1892).

